# Hidden Epidemic of Macrolide-resistant Pneumococci

**DOI:** 10.3201/eid1106.050147

**Published:** 2005-06

**Authors:** Keith P. Klugman, John R. Lonks

**Affiliations:** * Emory University, Atlanta, Georgia, USA;; † University of the Witwatersrand, Johannesburg, South Africa;; ‡ Brown Medical School, Providence, Rhode Island, USA

**Keywords:** Antibiotic resistance, Streptococcus pneumoniae, Macrolides, Community-acquired infections, Treatment failure

## Abstract

Community-acquired respiratory tract infections (RTIs) account for a substantial proportion of outpatient antimicrobial drug prescriptions worldwide. Concern over the emergence of multidrug resistance in pneumococci has largely been focused on penicillin-resistant *Streptococcus pneumoniae*. Macrolide antimicrobial drugs have been widely used to empirically treat community-acquired RTIs because of their efficacy in treating both common and atypical respiratory pathogens, including *S. pneumoniae*. However, increased macrolide use has been associated with a global increase in pneumococcal resistance, which is leading to concern over the continued clinical efficacy of the macrolides to treat community-acquired RTIs. We provide an overview of macrolide-resistant *S. pneumoniae* and assess the impact of this resistance on the empiric treatment of community-acquired RTIs.

Community-acquired respiratory tract infections (RTIs), including acute bacterial sinusitis, acute otitis media, acute exacerbations of chronic bronchitis, and community-acquired pneumonia, are among the most frequent infections treated by physicians and represent a major international health problem (1). Community-acquired pneumonia is one of the leading causes of hospitalization in the United States and the most common cause of death in patients with infectious diseases (2), while acute otitis media is the most frequent illness for which antimicrobial drugs are prescribed for children in the industrialized world. *Streptococcus pneumoniae* is the most common microbial pathogen identified in community-acquired RTIs, and pneumococcal infections are among the leading causes of illness and death worldwide (3), particularly among children, the elderly, and persons with coexisting medical conditions.

In the past, β-lactam antimicrobial drugs (e.g., penicillin) were widely used to empirically treat community-acquired RTIs. Pneumococcal resistance to penicillin was first observed in the 1960s; since then, the emergence and spread of penicillin-resistant *S. pneumoniae* strains have been observed and tracked worldwide. With the β-lactams in widespread use, increasing levels of penicillin-resistant *S. pneumoniae* were thought to be of greater potential clinical importance than the emergence of macrolide-resistant *S. pneumoniae* strains. However, a number of studies and analyses of patients with pneumococcal pneumonia (4) have shown no association between penicillin resistance and patient death, although some studies have indicated that penicillin-resistant *S. pneumoniae* infection may be associated with an increased risk for suppurative complications, longer hospital stays, and higher treatment costs (5).

The growing concerns about the emergence and spread of drug-resistant pathogens (including penicillin-resistant *S. pneumoniae*) and an increased awareness of infection with atypical pathogens (e.g., *Chlamydia pneumoniae*, *Mycoplasma pneumoniae*, and *Legionella pneumophila*), led to the publication of community-acquired pneumonia treatment guidelines by the American Thoracic Society in 1993 (6). These guidelines included a recommendation that macrolide drugs be used as first-line empiric therapy for outpatients with community-acquired pneumonia. The macrolides have since been used extensively to treat community-acquired RTIs worldwide. However, increasing macrolide use has also been associated with an increase in pneumococcal resistance to these agents, and macrolide-resistant *S. pneumoniae* are now more common than penicillin-resistant *S. pneumoniae* in many parts of the world (7). We provide an overview of pneumococcal resistance to macrolides and assess the impact of macrolide-resistant *S. pneumoniae* on the empiric treatment of community-acquired RTIs.

## Macrolide Resistance

### Mechanisms of Resistance

Macrolides are microbiostatic agents that reversibly bind to the 23S ribosomal RNA in the 50S subunit of ribosomes and block protein synthesis (8). Two main macrolide resistance mechanisms have been identified in pneumococci: active efflux of the drug from the cell and target-site modification (8). Energy-dependent efflux of macrolides from target cells by a cell membrane transporter has been associated with the presence of *mef* genes. Recent work by Iannelli et al. (9) has implicated a second gene, *mat*(A), that encoded 2 ATP-binding domains, as a component of *mef*-mediated macrolide resistance in pneumococci. Irrespective of the identity of the gene responsible for macrolide efflux, *mef*(A)-positive *S. pneumoniae* strains displaying this macrolide efflux phenotype (termed the M phenotype) are resistant to 14- and 15-membered ring macrolides (but not lincosamides or streptogramins) and generally display a low level of in vitro resistance to these antimicrobial agents. However, data from surveillance studies suggest that erythromycin MICs for *mef*(A)-positive isolates may be increasing. MICs were 1–16 μg/mL for *mef*(A)-positive *S. pneumoniae* isolates collected from 1994 to 1995 (10), while results from a more recent study demonstrated an erythromycin MIC of 1 to >256 μg/mL (11).

The second major mechanism of macrolide resistance in streptococci, target-site modification, is predominantly encoded by the *erm*(B) gene, resulting in methylation of an adenine residue on the 23S rRNA by a methylase enzyme. This methylation blocks the binding of macrolide-lincosamide-streptogramin B antimicrobial drugs. Strains with the macrolide-lincosamide-streptogramin B phenotype generally show higher levels of in vitro resistance to macrolides compared to strains with the M phenotype (10).

Other target-site modifications occur rarely in clinical isolates of *S. pneumoniae*. These modifications include mutations that involve domain V of the 23S rRNA and genes encoding riboproteins L4 and L22 (12). Such mutations can confer resistance to macrolide-lincosamide-streptogramin B antimicrobial drugs and are associated with variable levels of in vitro resistance. Although the global prevalence of pneumococcal strains with macrolide resistance conferred by ribosomal gene mutations remains low (<2%), a study of macrolide resistance mechanisms among *S. pneumoniae* isolated in Canada from 1997 to 2003 indicated that the rate of resistance due to mechanisms other than efflux or ribosomal methylation increased from 1% in 1997 to 10% in 2003 (13).

### Macrolide Resistance Trends

A number of industry-sponsored global surveillance studies, such as the Alexander Project (GlaxoSmithKline) and PROTEKT (Prospective Resistant Organism Tracking and Epidemiology for the Ketolide Telithromycin) (sanofi-aventis), have been designed to define and monitor the prevalence and distribution of antimicrobial resistance among respiratory pathogens, detect new patterns of resistance, provide early warning of emerging resistance, and evaluate the effects of interventions aimed at reducing antimicrobial resistance. Results from the Alexander Project indicate that in 1996 and 1997 the global rate of pneumococcal macrolide resistance was16.5%–21.9% (14); by 1998–2000, the resistance rate had increased to 24.6% (15). Data reported after completion of the first year of the PROTEKT study (1999–2000 respiratory season) confirmed this high global incidence (31.0%) of pneumococcal macrolide resistance (7), with similar overall levels of resistance among isolates collected as part of the PROTEKT US study (31.0% in 2000–2001 and 27.9% in 2001–2002) (16,17). The slight reduction in macrolide resistance among pneumococcal isolates collected as part of the PROTEKT US study from 2001 to 2002 may be a consequence of the February 2000 introduction of the 7-valent pneumococcal conjugate vaccine (18).

However, both macrolide resistance rates and resistance mechanisms may vary considerably depending on location. Macrolide resistance rates for isolates collected during the PROTEKT US study from 2001 to 2002 vary according to region; at a state level, the highest prevalence of pneumococcal macrolide resistance was recorded in Louisiana (48.2%) and the lowest in Vermont (15.2%) (11). Similarly, while *mef*(A) was the most prevalent pneumococcal macrolide resistance genotype identified in the United States overall (68.7% of genotyped isolates), the relative prevalence varied by state and ranged from 40% in Delaware to 85% in Georgia (11). While *erm*(B) was the second most prevalent genotype overall (16.8%), isolates possessing both the *erm*(B) and *mef*(A) genotype (12.2%) were more prevalent in 11 states than those harboring *erm*(B) alone. A recent analysis of PROTEKT US 2002–2003 data by Farrell et al. (19) indicates an increase in the prevalence of macrolide-resistant isolates containing both *erm*(B) and *mef*(A) from 9.7% in 2000–2001 to 16.4% in 2002–2003. Most (99.2%) of these *erm*(B) + *mef*(A)–positive isolates were resistant to ≥2 classes of antimicrobial drugs. Analysis of erythromycin MIC data for all macrolide-resistant isolates collected in 2000–2001 indicated that the MIC_90_ (MIC at which 90% of isolates were inhibited) varied according to resistance genotype (16 μg/mL for *mef*(A)-positive isolates vs. >256 μg/mL for *erm*(B)-positive isolates and those harboring both the *erm*(B) and *mef*(A) gene) (11).

### Factors Contributing to Development and Spread of Macrolide Resistance

Inappropriate use of antimicrobial drugs is among the most important factors associated with the emergence and spread of pneumococcal macrolide resistance. Inappropriate use may include using antimicrobial drugs to treat nonmicrobial or self-limiting infections, using agents with a spectrum of activity that either does not cover the appropriate causative pathogen(s) or which has too broad a spectrum of activity, and inappropriate dose or duration of treatment (20).

Other risk factors for carriage or infection with resistant pneumococcal strains include age (patients particularly at risk include those <2 or >65 years of age), history of macrolide use, and the presence of severe underlying disease (21). Analyses of data from national and international surveillance studies have suggested a link between increased use of macrolides and increased rates of pneumococcal resistance (22). In Portugal, the emergence of macrolide-resistant *S. pneumoniae* strains from 1994 to 2002 correlated with the use of azithromycin during the same period (23). Several studies have shown that macrolide administration is associated with increased nasopharyngeal carriage of resistant strains of *S. pneumoniae* in children (24); the clonal dissemination of macrolide-resistant pneumococcal strains in crowded environments (e.g., daycare centers, hospitals, jails, long-term care facilities) is also thought to be a major factor contributing to the spread of resistance.

### Clinical Implications of Macrolide Resistance

Surveillance studies have shown that a substantial percentage of pneumococci are now macrolide resistant. Despite this rising rate of in vitro resistance, some researchers and clinicians have questioned whether resistance to macrolides is clinically relevant given the high concentrations achieved in respiratory tissues such as the epithelial lining fluid. Although macrolide levels in epithelial lining fluid have been reported to exceed the levels achieved in serum, the relevance of the fluid levels has been questioned (25), and sufficiently high macrolide blood levels remain essential to cure bacteremic pneumococcal pneumonia. Moreover, clinically achievable serum, epithelial lining fluid, and middle-ear fluid concentrations of azithromycin were insufficient to eradicate macrolide-resistant *S. pneumoniae*, irrespective of the resistance mechanism (26).

Prospective clinical studies have provided conflicting evidence for an association between discordant antimicrobial therapy (i.e., use of an agent to which the causative pathogen displayed in vitro resistance) and treatment outcome. For example, while results from 1 study of patients with community-acquired pneumonia and bacteremia due to bacteremic pneumococcal infection demonstrated an association between increased death rates and discordant antimicrobial drug therapy (27), no such association was observed in a different study (28) of patients with pneumococcal community-acquired pneumonia. However, the conclusions that can be drawn from such studies may be limited by factors such as small sample size, differences in patient inclusion or exclusion criteria (e.g., recent antimicrobial drug use), use of relatively insensitive measures of treatment outcome (e.g., death), and use of single or multiple antimicrobial drugs (many hospitalized patients receive combination antimicrobial therapy, thus limiting the opportunities to study the effects of discordant treatment on clinical outcomes); in the studies cited above, none of the cases of discordant therapy involved monotherapy with a macrolide.

In acute otitis media, tympanocentesis performed before and after drug therapy has been used in several studies to determine the clinical relevance of antimicrobial resistance. Using this method, Dagan et al. (29) showed that microbiologic failure (correlated with clinical failure) was associated with pneumococcal macrolide resistance among patients treated with azithromycin; treatment of 6 of 6 patients with high-level macrolide resistance failed microbiologically. Furthermore, analysis of data from a pediatric medical center in the United States (30) noted that the rising incidence of antimicrobial-resistant pneumococci corresponded to an increase in suppurative complications of acute otitis media and appeared to contribute to more aggressive infections that required surgical intervention.

In recent years, several reports have described clinical and microbiologic treatment failures that have occurred in hospitalized patients infected with macrolide-resistant pneumococci (31). Among these cases of treatment failure, 2 deaths occurred. In both cases, the previously healthy patients (a 28-year-old man and a 49-year-old woman) received monotherapy with intravenous azithromycin for pneumonia. The clinical status of both patients deteriorated while they were receiving azithromycin, and macrolide-resistant *S. pneumoniae* were isolated from blood and pleural fluid cultures taken while these patients were receiving medication.

A matched case-control study of hospitalized patients with bacteremia conducted by Lonks et al. (32) identified 86 patients with isolates of *S. pneumoniae* that were fully or intermediately resistant to macrolides and 141 controls who had macrolide-susceptible pneumococcal infection. When patients with meningitis were excluded from the analysis, 18 (24%) of 76 patients were taking a macrolide at the time of bacteremia compared to none of the controls (p<0.0001). Moreover, 5 (24%) of the 21 bacteremic patients infected with pneumococci expressing the M phenotype were taking a macrolide (compared with none of the 40 matched control patients; p<0.0016). These data show that breakthrough bacteremia and treatment failure occurred only in those patients infected with a macrolide-resistant pneumococcus; no incidences of breakthrough bacteremia were seen in those infected with a macrolide-susceptible pneumococcus. Similarly, a study of all pneumococcal bacteremias from a hospital in Belgium (33) showed that 4 (12%) of 33 patients with a macrolide-resistant pneumococcus were taking a macrolide when blood cultures were obtained, i.e., they had breakthrough bacteremia; in contrast, none of the 103 patients with macrolide-susceptible pneumococci was taking a macrolide.

The overall incidence of treatment failure caused by macrolide-resistant pneumococci cannot be estimated from the case reports and observational studies published to date. These reports of treatment failure likely only represent the tip of the iceberg, as most case studies published to date have only captured treatment failures that resulted in breakthrough bacteremia. These published studies underreport the magnitude of treatment failures because nonbacteremic pneumococcal pneumonia is 3–5 times more common than bacteremic pneumonia. In addition, these treatment failures resulted in hospitalization, which is more expensive than outpatient therapy. Most macrolides are prescribed as part of empiric treatment regimens for ambulatory patients in the outpatient setting; microbiologic cultures are not usually obtained from these patients, and antimicrobial susceptibility testing is rarely performed (even if treatment failure occurs).

### Macrolide Resistance and Treatment Guidelines

In the United States, guidelines for the treatment of community-acquired RTIs have been established by a number of groups, including the American Thoracic Society, the Infectious Diseases Society of America, the Centers for Disease Control and Prevention (CDC), and the Sinus and Allergy Health Partnership. The clinical relevance of macrolide-resistant *S. pneumoniae* has been addressed in updates to these groups' guidelines for the treatment of community-acquired pneumonia (34,35) and in a report published by the Drug-Resistant *Streptococcus pneumoniae* Therapeutic Working Group convened by CDC (36). The consensus among these guidelines is that empiric therapy should be stratified based on likely cause, treatment setting (inpatient versus outpatient), and the risk for pneumococcal antimicrobial resistance. In general, all 3 guidelines recommend that monotherapy with macrolides should be restricted to specific patient subgroups (i.e., those with no coexisting cardiopulmonary disease and no risk factors for infection with drug-resistant *S. pneumoniae* [e.g., recent antimicrobial drug use]). For outpatients with risk factors for drug-resistant *S. pneumoniae*, current recommended treatment options include combination therapy with a β-lactam (such as high-dose amoxicillin or high-dose amoxicillin-clavulanate) plus a macrolide or an antipneumococcal fluoroquinolone (34,35). The increased use of fluoroquinolones has been associated with the emergence and spread of resistance to these agents (37), and local clonal dissemination of *S. pneumoniae* strains with very high-level resistance to penicillin has been reported in the United States (38). Although the prevalence of these resistant isolates remains low, such findings emphasize the necessity for local resistance patterns to be considered when prescribing empiric antimicrobial drug therapy for patients with community-acquired RTIs.

The Sinus and Allergy Health Partnership guidelines for the treatment of acute microbial rhinosinusitis also highlight the need to consider the increasing prevalence of pneumococcal resistance when making treatment choices, with patients divided into categories dependent on their recent exposure to antimicrobial drugs (39). Similarly, a recent American Thoracic Society statement on the management of acute microbial exacerbations of chronic obstructive pulmonary disease emphasizes the need to consider local resistance patterns when prescribing antimicrobial drugs (40).

## Conclusions

National and international surveillance studies demonstrate a high global prevalence of in vitro macrolide resistance among pneumococcal isolates obtained from patients with community-acquired RTIs. In recent years, a number of studies have clearly linked in vitro macrolide resistance to microbiologic and clinical treatment failure, indicating that macrolide resistance is an emerging problem. As pneumococcal community-acquired RTIs (particularly community-acquired pneumonia) are a leading cause of illness and death worldwide, appropriate empiric antimicrobial therapy should be used to treat these infections. Recent updates to a number of treatment guidelines have reflected this changing situation by emphasizing the need for clinicians to consider local antimicrobial resistance patterns and risk factors for infection with drug-resistant pathogens when prescribing empiric antimicrobial therapy.

## References

[1] World Health Organization. The World health report 2004 — changing history [monograph on the Internet]. 2004 [cited 2005 Apr 1]. Available from http://www.who.int/whr/2004/en/report04_en.pdf

[2] Hall MJ, DeFrances CJ. National hospital discharge survey. Adv Data. 2002;332:1–18. 15174387

[3] Jacobs MR. *Streptococcus pneumoniae*: epidemiology and patterns of resistance. Am J Med. 2004;117(Suppl 3A):38–15S. 15360092 10.1016/j.amjmed.2004.07.003

[4] Klugman KP. Bacteriological evidence of antibiotic failure in pneumococcal lower respiratory tract infections. Eur Respir J. 2002;36(Suppl):36:3–8s. 10.1183/09031936.02.00400402 12168746

[5] Metlay JP, Hofmann J, Cetron MS, Fine MJ, Farley MM, Whitney C, Impact of penicillin susceptibility on medical outcomes for adult patients with bacteremic pneumococcal pneumonia. Clin Infect Dis. 2000;30:520–8. 10.1086/313716 10722438

[6] Niederman MS, Bass JB Jr, Campbell GD, Fein AM, Grossman RF, Mandell LA, Guidelines for the initial management of adults with community-acquired pneumonia: diagnosis, assessment of severity, and initial antimicrobial therapy. Am Rev Respir Dis. 1993;148:1418–26. 8239186 10.1164/ajrccm/148.5.1418

[7] Felmingham D, Reinert RR, Hirakata Y, Rodloff A. Increasing prevalence of antimicrobial resistance among isolates of *Streptococcus pneumoniae* from the PROTEKT surveillance study, and comparative in vitro activity of the ketolide, telithromycin. J Antimicrob Chemother. 2002;50(Suppl 2):25–37. 10.1093/jac/dkf808 12239226

[8] Zhanel GG, Dueck M, Hoban DJ, Vercaigne LM, Embil JM, Gin AS, Review of macrolides and ketolides: focus on respiratory tract infections. Drugs. 2001;61:443–98. 10.2165/00003495-200161040-00003 11324679

[9] Iannelli F, Santagat M, Doquier JD, Cassone M, Oggioni MR, Rossolini G, Type M resistance to macrolides in streptococci is not due to the *mef*(A) gene, but to *mat*(A) encoding and ATP-dependent efflux pump [Abstract C1-1188]. In: Abstracts of the 44th Interscience Conference on Antimicrobial Agents and Chemotherapy; Washington; 2004 Oct 30–Nov 2.

[10] Shortridge VD, Doern GV, Brueggemann AB, Beyer JM, Flamm RK. Prevalence of macrolide resistance mechanisms in *Streptococcus pneumoniae* isolates from a multicenter antibiotic resistance surveillance study conducted in the United States in 1994–1995. Clin Infect Dis. 1999;29:1186–8. 10.1086/313452 10524961

[11] Farrell DJ, Jenkins SG. Distribution across the USA of macrolide resistance and macrolide resistance mechanisms among *Streptococcus pneumoniae* isolates collected from patients with respiratory tract infections: PROTEKT US 2001–2002. J Antimicrob Chemother. 2004;54(Suppl 1):i17–22. 10.1093/jac/dkh312 15265832

[12] Tait-Kamradt A, Davies T, Cronan M, Jacobs MR, Appelbaum PC, Sutcliffe J. Mutations in 23S rRNA and ribosomal protein L4 account for resistance in pneumococcal strains selected in vitro by macrolide passage. Antimicrob Agents Chemother. 2000;44:2118–25. 10.1128/AAC.44.8.2118-2125.2000 10898684 PMC90022

[13] Wierzbowski AK, Swedlo D, Nichol K, Hisanaga T, Rusen J, Hoban D, Resistance gentoypes among macrolide resistant *Streptococcus pneumoniae* isolated in Canada between 1997 and 2003 [Abstract C2-821]. In: Abstracts of the 44th Interscience Conference on Antimicrobial Agents and Chemotherapy; Washington; 2004 Oct 30–Nov 2.

[14] Felmingham D, Grüneberg RN. The Alexander Project 1996–1997: latest susceptibility data from this international study of bacterial pathogens from community-acquired lower respiratory tract infections. J Antimicrob Chemother. 2000;45:191–203. 10.1093/jac/45.2.191 10660501

[15] Jacobs MR, Felmingham D, Appelbaum PC, Gruneberg RN; Alexander Project Group. The Alexander Project 1998–2000: susceptibility of pathogens isolated from community-acquired respiratory tract infection to commonly used antimicrobial agents. J Antimicrob Chemother. 2003;52:229–46. 10.1093/jac/dkg321 12865398

[16] Doern GV, Brown SD. Antimicrobial susceptibility among community-acquired respiratory tract pathogens in the USA: data from PROTEKT US 2000–01. J Infect. 2004;48:56–65. 10.1016/S0163-4453(03)00123-3 14667792

[17] Brown SD, Rybak MJ. Antimicrobial susceptibility of *Streptococcus pneumoniae*, *Streptococcus pyogenes* and *Haemophilus influenzae* collected from patients across the USA, in 2001–2002, as part of the PROTEKT US study. J Antimicrob Chemother. 2004;54(Suppl 1):i7–15. 10.1093/jac/dkh313 15265831

[18] Whitney CG, Farley MM, Hadler J, Harrison LH, Bennett NM, Lynfield R, Decline in invasive pneumococcal disease after the introduction of protein-polysaccharide conjugate vaccine. N Engl J Med. 2003;348:1737–46. 10.1056/NEJMoa022823 12724479

[19] Farrell D, Jenkins S, Brown S, Patel M, Lavin B, Klugman K. Emergence and spread of *Streptococcus pneumoniae* with *erm*(B) and *mef*(A) resistance. Emerg Infect Dis. 2005;11:851–8. 15963279 10.3201/eid1106.050222 PMC3367592

[20] Lieberman JM. Appropriate antibiotic use and why it is important: the challenges of bacterial resistance. Pediatr Infect Dis J. 2003;22:1143–51. 10.1097/01.inf.0000101851.57263.63 14688589

[21] Jacobs MR. Drug-resistant *Streptococcus pneumoniae*: rational antibiotic choices. Am J Med. 1999;106:19–25. 10.1016/S0002-9343(98)00351-9 10348060

[22] Baquero F. Evolving resistance patterns of *Streptococcus pneumoniae*: a link with long-acting macrolide consumption? J Chemother. 1999;11(Suppl 1):35–43. 10207772 10.1179/joc.1999.11.Supplement-2.35

[23] Dias R, Caniça M. Emergence of invasive erythromycin-resistant *Streptococcus pneumoniae* strains in Portugal: contribution and phylogenetic relatedness of serotype 14. J Antimicrob Chemother. 2004;54:1035–9. 10.1093/jac/dkh469 15531597

[24] Leach AJ, Shelby-James TM, Mayo M, Gratten M, Laming AC, Currie BJ, A prospective study of the impact of community-based azithromycin treatment of trachoma on carriage and resistance of *Streptococcus pneumoniae*. Clin Infect Dis. 1997;24:356–62. 10.1093/clinids/24.3.356 9114185

[25] Jacobs MR. In vivo veritas: in vitro macrolide resistance in systemic *Streptococcus pneumoniae* infections does result in clinical failure. Clin Infect Dis. 2002;35:565–9. 10.1086/341980 12173130

[26] Zhanel GG, DeCorby M, Noreddin A, Mendoza C, Cumming A, Nichol K, *Streptococcus pneumoniae* simulating clinically achievable free serum, epithelial lining fluid and middle ear fluid concentrations. J Antimicrob Chemother. 2003;52:83–8. 10.1093/jac/dkg278 12775677

[27] Lujan M, Gallego M, Fontanals D, Mariscal D, Rello J. Prospective observational study of bacteremic pneumococcal pneumonia: effect of discordant therapy on mortality. Crit Care Med. 2004;32:625–31. 10.1097/01.CCM.0000114817.58194.BF 15090938

[28] Ewig S, Ruiz M, Torres A, Marco F, Martinez JA, Sanchez M, Pneumonia acquired in the community through drug-resistant *Streptococcus pneumoniae*. Am J Respir Crit Care Med. 1999;159:1835–42. 10351928 10.1164/ajrccm.159.6.9808049

[29] Dagan R, Leibovitz E, Fliss DM, Leiberman A, Jacobs MR, Craig W, Bacteriologic efficacies of oral azithromycin and oral cefaclor in treatment of in infants and young children. Antimicrob Agents Chemother. 2000;44:43–50. 10.1128/AAC.44.1.43-50.2000 10602721 PMC89626

[30] Zapalac JS, Billings KR, Schwade ND, Roland PS. Suppurative complications of |in the era of antibiotic resistance. Arch Otolaryngol Head Neck Surg. 2002;128:660–3. 12049560 10.1001/archotol.128.6.660

[31] Lonks JR. What is the clinical impact of macrolide resistance? Curr Infect Dis Rep. 2004;6:7–12. 10.1007/s11908-004-0018-1 14733843

[32] Lonks JR, Garau J, Gomez L, Xercavins M, Ochoa de Echaguen A, Gareen IF, Failure of macrolide antibiotic treatment in patients with bacteremia due to erythromycin-resistant *Streptococcus pneumoniae*. Clin Infect Dis. 2002;35:556–64. 10.1086/341978 12173129

[33] Van Kerkhoven D, Peetermans WE, Verbist L, Verhaegen J. Breakthrough pneumococcal bacteraemia in patients treated with clarithromycin or oral beta-lactams. J Antimicrob Chemother. 2003;51:691–6. 10.1093/jac/dkg116 12615872

[34] Niederman MS, Mandell LA, Anzueto A, Bass JB, Broughton WA, Campbell GD, Guidelines for the management of adults with community-acquired pneumonia. Diagnosis, assessment of severity, antimicrobial therapy, and prevention. Am J Respir Crit Care Med. 2001;163:1730–54. 11401897 10.1164/ajrccm.163.7.at1010

[35] Mandell LA, Bartlett JG, Dowell SF, File TM Jr, Musher DM, Whitney C. Update of practice guidelines for the management of community-acquired pneumonia in immunocompetent adults. Clin Infect Dis. 2003;37:1405–33. 10.1086/380488 14614663 PMC7199894

[36] Heffelfinger JD, Dowell SF, Jorgensen JH, Klugman KP, Mabry LR, Musher DM, Management of community-acquired pneumonia in the era of pneumococcal resistance: a report from the Drug-Resistant *Streptococcus pneumoniae* Therapeutic Working Group. Arch Intern Med. 2000;160:1399–408. 10.1001/archinte.160.10.1399 10826451

[37] Goldstein EJ, Garabedian-Ruffalo SM. Widespread use of fluoroquinolones versus emerging resistance in pneumococci. Clin Infect Dis. 2002;35:1505–11. 10.1086/344768 12471570

[38] Schrag SJ, McGee L, Whitney CG, Beall B, Craig AS, Choate ME, Emergence of *Streptococcus pneumoniae* with very high-level resistance to penicillin. Antimicrob Agents Chemother. 2004;48:3016–23. 10.1128/AAC.48.8.3016-3023.2004 15273115 PMC478489

[39] Anon JB, Jacobs MR, Poole MD, Ambrose PG, Benninger MS, Hadley JA, Antimicrobial treatment guidelines for acute bacterial rhinosinusitis. Otolaryngol Head Neck Surg. 2004;130(Suppl):1–45. 14726904 10.1016/j.otohns.2003.12.003 PMC7118847

[40] The American Thoracic Society and the European Respiratory Society. Standards for the diagnosis and management of patients with COPD (2004). [cited 2005 Apr 1]. Available from http://www.thoracic.org/COPD

